# Idiopathic Epicardial Ventricular Arrhythmias: Diagnosis and Ablation Technique from the Aortic Sinus of Valsalva

**Published:** 2005-04-01

**Authors:** Hiroshi Tada

**Affiliations:** Division of Cardiology, Gunma Prefectural Cardiovascular Center, Maebashi, Gunma Japan

**Keywords:** Ventricular tachycardia, left sinus of Valsalva, potential, premature ventricular contraction, catheter ablation, tissue tracking imaging

## Abstract

Idiopathic outflow tract arrhythmias (ventricular tachycardias or symptomatic premature ventricular contractions; OT-VT/PVCs) can originate from the left ventricular (LV) epicardium (Epi-VT/PVCs), and radiofrequency (RF) energy applications from the aortic sinus of Valsalva can eliminate Epi-VT/PVCs in selected patients. Among the various ECG findings, the R-wave duration index and R/S amplitude index in leads V1 or V2 are useful for identifying Epi-VT/PVCs, and the Q-wave ratio of leads aVL to aVR and S-wave amplitude in lead V1 are useful for differentiating between an Epi-VT/PVC originating from the LV epicardium remote from the left sinus of Valsalva (LSV) and that from the LSV. Tissue tracking imaging is a promising modality for identifying the origin of OT-VT/PVCs and for differentiating between an Epi-VT/PVC originating from the LV epicardium remote from the LSV and that from the LSV.

If the origin of the Epi-VT/PVC is identified within the LSV, coronary and aortic angiography should be performed to assess the anatomic relationships between the Epi-VT/PVC origin and coronary arteries and aortic valve before the RF energy delivery. To avoid potential complications, RF ablation should be performed at the LSV using a maximum power of 35 watts and maximum temperature of 55°C. Epicardial mapping through the coronary venous system and the presence of potentials recorded from the ablation site within the LSV and their changes before and after the RF energy applications may be useful for diagnosing Epi-VT/PVCs or predicting a successful catheter ablation from the LSV.

## Introduction

Radiofrequency (RF) catheter ablation has been established as an effective and curative therapy for ventricular tachycardias (VTs) or symptomatic premature ventricular contractions (PVCs) originating from the outflow tract (OT-VT/PVCs) in structurally normal hearts [1-6]. Recenty, idiopathic OT-VT/PVCs originating from the left ventricular (LV) epicardium (Epi-VT/PVCs) have been reported [6-13]. These OT-VT/PVCs are thought to originate from the LV epicardium around the transitional area from the great cardiac vein to the anterior interventricular vein [13,14]. Some can be ablated through the coronary venous system [15] or by percutaneous epicardial instrumentation [16], but others can be ablated from the left sinus of Valsalva (LSV) [6-13]. The correct identification of the latter type of OT-VT/PVC before the RF energy applications is important in order to avoid futile RF applications from the LSV and the ensuing complications [17,18]. In this article, we focused on the diagnosis and RF catheter ablation of Epi-VT/PVCs which can be ablated from the LSV.

## Diagnosis

**Figure 1:**
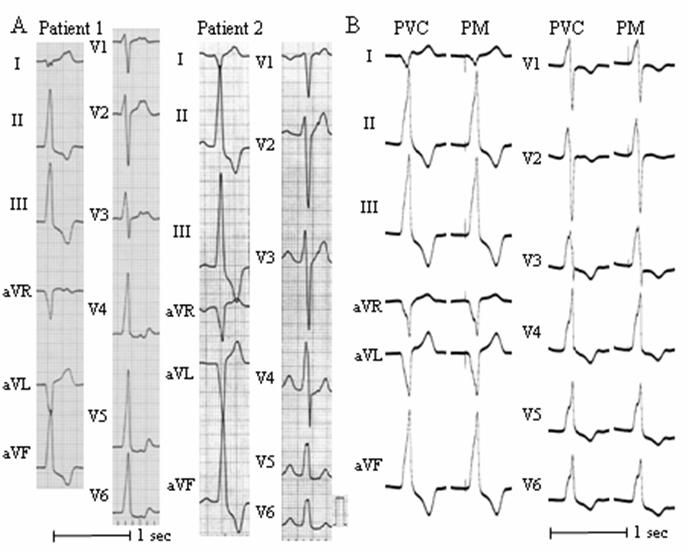
A. Representative 12-lead ECGs of premature ventricular contractions (PVCs) originating from the left sinus of Valsalva (LSV; Patient 1) and left ventricular (LV) epicardium remote from the LSV (Patient 2). Both PVCs have an R-wave duration index in lead V2 ≥0.5, indicating that these PVCs originate from the LV epicardium. The Q-wave ratio of aVL to aVR was ≤1.4 and S-wave amplitude <1.2 mV in lead V1 in patient 1, and the Q-wave ratio of aVL to aVR was >1.4 and S-wave amplitude ≥1.2 mV in patient 2. The former PVC could be eliminated by an RF energy application from the LSV, but the latter could not be ablated from the LSV. B. Twelve-lead ECG of a PVC and pace mapping (PM) from the LSV. The configuration of the 12-lead QRS complex that was induced by stimulation through the ablation catheter within the LSV matched that of the spontaneous PVC. An RF application at this site eliminated this PVC.

Characteristic ECG findings originating from each portion of the outflow tract and several ECG findings useful for differentiating an OT-VT/PVC from other OT-VT/PVCs originating from different portions of the outflow tract have been reported. The precordial R-wave transition [3,6,19], QRS morphology in lead I [3,6], R-wave duration in lead V1 or V2, and R/S-wave amplitude ratio in leads V1 or V2 have been reported as useful indices for differentiating OT-VT/PVCs originating from the right side from those arising from the left side (LVOT, LSV or LV epicardium remote from the LSV) [3,6,13]. Furthermore, the presence of an S wave in lead V5 or V6 is considered a characteristic, useful ECG finding in OT-VT/PVCs of LVOT origin [6,9]. Among those findings, the usefulness of both the R-wave duration index and R/S amplitude index in leads V1 or V2 for identifying Epi-VT/PVCs has been reported (Figure 1) [6-8]. The R-wave duration index is obtained by dividing the QRS complex duration by the longer R-wave duration in lead V1 or V2, and the R/S-wave amplitude index is calculated as the greater value for the R/S-wave amplitude ratio in lead V1 or V2 [6,8]. A recent study demonstrated that when the R-wave duration index is ≥0.5 or the R/S-wave amplitude index is ≥0.3, there is a fair possibility that the OT-VT/PVC originates from the LSV (Figure 1A, left panel) or LV epicardium remote from the LSV which cannot be ablated from the LSV (Fig. 1A, right panel) [6]. However, both indices do not significantly differ when differentiating between Epi-VT/PVCs originating from the LSV and those originating from the LV epicardium remote from the LSV [6]. Instead, a Q-wave ratio of leads aVL to aVR >1.4 or an S-wave amplitude ≥1.2 mV in lead V1 was useful for differentiating between an Epi-VT/PVC originating from the LV epicardium remote from the LSV and that from the LSV (Figure 1) [6]. In a previous report using a pace mapping techinique [13], the QRS morphology during pacing from the proximal site of the anterior interventricular vein (AIV) exhibited a deep S-wave in V1, and pacing from the distal portion of the great cardiac vein (GCV) revealed a high Q-wave ratio of aVL to aVR. Therefore, a high Q-wave ratio for aVL to aVR or a deep S-wave in lead V1 may indicate that the Epi-VT/PVC originates from the LV epicardium remote from the LSV around the transitional area from the GCV to the AIV [13,14]. For determination of the location of OT-VT/PVCs, including Epi-VT/PVCs, a recently developed ECG algorithm is useful [6].

Although rare (3%), some OT-VT/PVCs have demonstrated dynamic changes in the QRS morphology following the RF catheter ablation, requiring additional RF ablation applications at a different portion of the outflow tract to cure the OT-VT/PVCs [20]. These OT-VT/PVCs consistently showed a change in the R wave amplitude in the inferior leads between the 1^st^ OT-VT/PVC and 2nd OT-VT/PVC, while the earliest ventricular activation during either the 1^st^ or 2^nd^ OT-VT/PVC was recorded from the LSV [20]. When an increase in the R-wave amplitude in the inferior leads follows the RF catheter ablation of the 1st OT-VT/PVC from an endocardial site of the RVOT or LVOT, the earliest ventricular activation during the 2^nd^ OT-VT/PVC may be recorded at the LSV. On the other hand, when a decrease in the R-wave amplitude in the inferior leads follows the RF catheter ablation from the LSV, the earliest ventricular activation during the 2^nd^ OT-VT may be recorded from an endocardial site of the RVOT or LVOT. Therefore, detailed continuous observation of the QRS morphology of OT-VT/PVCs, especially the R wave amplitude in the inferior leads, is important for identifying the changes in the QRS morphology during the RF catheter ablation of OT-VT/PVCs.

**Figure 2:**
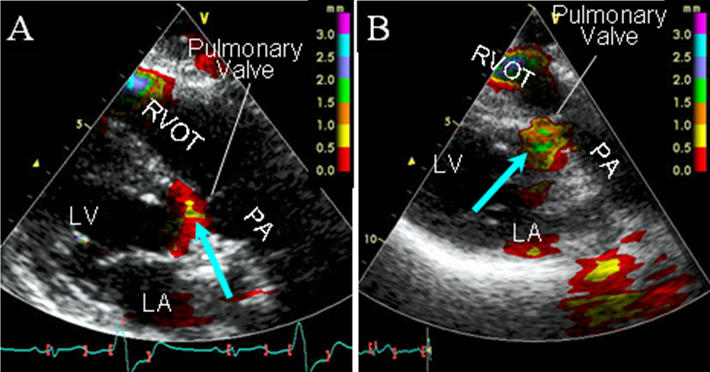
Tissue tracking images obtained from 2 patients with idiopathic premature ventricular contractions (PVC) originating from the outflow tract. A. The earliest color-coded signal (ECCS; blue arrow) appeared at the level of the pulmonary valve during a PVC. This PVC was successfully eliminated by an RF energy application to the septum of the right ventricular outflow tract (RVOT) just beneath the pulmonary valve. B. The ECCS (blue arrow) appeared in the myocardium 8 mm above the pulmonary valve during a PVC. This PVC was successfully ablated from the left sinus of Valsalva. LA= left atrium; LV= left ventricle; PA= pulmonary artery.

Recently, tissue tracking imaging (TTI) has been demonstrated as a novel non-invasive modality for identifying the origin of OT-VT/PVCs [21]. TTI is an ultrasonographic technique that measures the myocardial motion amplitude toward the transducer in each region during systole, identifying regional myocardial displacement on the basis of myocardial velocities using color Doppler myocardial imaging principles [22,23]. It allows rapid semiquantitative visual assessment of the systolic distance of the tissue motion along the Doppler axis using a graded color display [21-23]. In this technique, the origin of the OT-VT/PVC could be recognized as the site where the earliest color-coded signal (ECCS) appeared on the myocardium at the onset of the OT-VT/PVC (Figure 2) [21]. The OT-VT/PVCs in which the earliest ventricular activation was recorded from the LSV had the ECCS in the myocardium above the pulmonary valve, and most of them in which the ECCS appeared close to the pulmonary valve could be ablated from the LSV (Figure 2B) [21]. The distance between the attachment of the pulmonary valve to the septum and the center of the ECCS (8±4 mm) in these OT-VT/PVCs with a successful ablation from the LSV was significantly shorter than that in those with a failed ablation (18±6 mm, p<0.05). On the other hand, the ECCS was always found below or at the level of the pulmonary valve in all arrhythmias which could be ablated from the RVOT(Figure 2A). These results indicate that TTI can provide detailed and accurate information on the arrhythmia origin of OT-VT/PVCs and may be useful for differentiating between an OT-VT/PVC originating from the LV epicardium remote from the LSV and that from the LSV.

## Mapping and Ablation Technique

**Figure 3:**
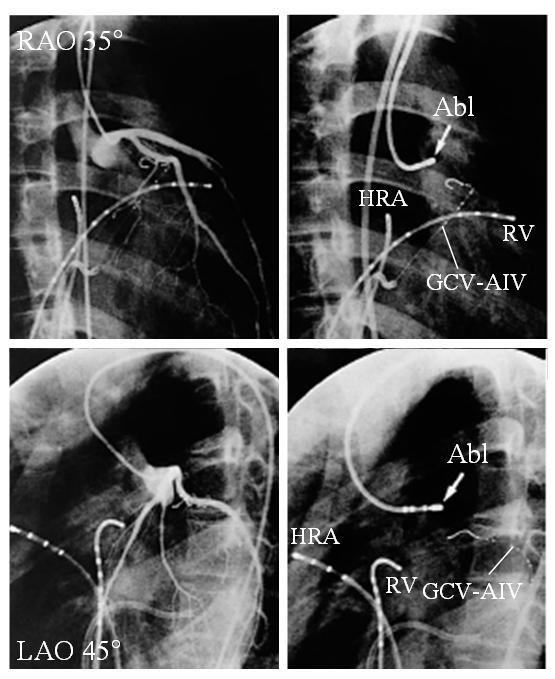
Left coronary artery (left) and fluoroscopic images during the radiofrequency catheter ablation (right) obtained in the right anterior oblique (RAO 35°) and left anterior oblique (LAO 45°) projections showing the ablation sites. The tip of the catheter at the successful ablation sites (Abl) was located about 10 mm below the left coronary artery ostium. An RF energy delivery at this site terminated the tachycardia. GCV-AIV=2-Fr octapolar electrode catheter placed from the great cardiac vein and anterior interventricular vein; HRA=high right atrium; RV= right ventricle.

Under guidance with fluoroscopy, catheters were introduced into the RV apex, RVOT and/or His bundle region via the right femoral vein. If the clinical arrhythmia did not occur spontaneously, programmed ventricular stimulation from the RV apex and RVOT or incremental burst pacing were performed. If the clinical arrhythmia was not induced in the baseline state, intravenous isoproterenol (0.5 to 2.0 μg/min) was administered to induce the clinical arrhythmia. Activation mapping and pace mapping were performed during the clinical arrhythmia. Mapping of the OT-VT/PVC was initially started in the RVOT region. If suitable ablation sites were not found in the RVOT, the endocardium of the LVOT and aortic sinus of Valsalva were mapped. If the origin of the OT-VT/PVC was mapped to a location above the aortic valve, coronary and aortic angiography were performed to assess the anatomic relationship between the OT-VT/PVC origin and coronary arteries and aortic valve before the RF energy delivery (Figure 3) [6-9,12,13].

It is believed that an RF application from the LSV does not ablate the valve or aortic wall itself, but destroys the epicardium above the septum [9]. In our laboratory, a 7 Fr quadripolar catheter with a 4-mm distal electrode, embedded thermistor, interelectrode spacing of 2-5-2 mm, and deflectable tip was usually used for mapping and ablation (Figure 3). To avoid potential complications, the RF ablation was performed at the aortic sinus of Valsalva using a maximum power of 35 watts, maximum electrode-tissue interface temperature of 55°C and maximum of 6 RF applications [9]. The applications of RF energy were 60 to 90 sec in duration. If an application of RF energy was unsuccessful, the ablation site was changed under fluoroscopic guidance. Pace mapping from the LSV was possible (Figure 1B), and several studies reported that a perfect pace mapping could be obtained from the successful ablation site [9,13]. However, the pacing threshold was usually too high (>8 V) to capture the myocardium through the LSV [9], and no studies have sufficiently examined whether or not an RF energy application at a site where a perfect pace map is obtained can always result in successful ablation, or whether or not an RF energy application at a site where a perfect pace map cannot be obtained results in a failed ablation.

### Utility of Epicardial Mapping through the Coronary Venous System

It is well known that even if the earliest ventricular activation of the Epi-VT/PVC is at the LSV, RF catheter ablation at that site is not always effective. Because the tip temperature should be maintained at <55°C to avoid potential complications [9], the distance between the ablation site within the LSV and origin of the Epi-VT/PVC will mainly affect the results of the RF ablation from the LSV. Therefore, some Epi-VT/PVCs with the earliest ventricular activation in the LSV cannot be ablated from the LSV because of the distance from the LSV. No criterion based on the electrograms for predicting a successful catheter ablation from the LSV has been established. However, when the earliest ventricular activation during the clinical arrhythmia was found within the LSV, the placement of a 2-Fr octapolar electrode catheter from the GCV to the AIV (GCV-AIV) and obtaining 7 bipolar electrogram recordings from the adjacent electrodes of the catheter placed along the GCV-AIV may be helpful in diagnosing Epi-VT/PVCs and predicting the successful RF catheter ablation from the LSV [24]. In Epi-VT/PVCs, the earliest ventricular activation during the arrhythmias is usually found at the LSV or GCV-AIV [13,14]. Therefore, several studies have attempted to evaluate the utility of epicardial mapping through the coronary venous system for diagnosising and treating Epi-VT/PVCs [6,8,9,13]. However, their results differ. Kanagaratnam et al. reported simultaneous endocardial and percutaneous epicardial mapping in patients with Epi-VTs eliminated by an RF application from the LSV [7]. They found that the Epi-VT/PVC had an earlier activation on the epicardial surface than at the endocardial site [7]. However, Hachiya et al. reported that the ventricular activation recorded from the LSV must be earlier than the earliest ventricular activation recorded in the GCV-AIV in Epi-VT/PVCs which can be ablated from the LSV [9]. A recent study reported the usefulness of comparing the earliness of the ventricular activation recorded from the LSV and earliest ventricular activation of 7 bipolar recordings from the GCV-AIV during the Epi-VT/PVCs for predicting a successful catheter ablation from the LSV [24]. In patients in whom the earliest ventricular activation was found at the LSV or GCV-AIV, the earliest ventricular activation in the GCV-AIV preceding the ventricular activation from the LSV by less than 10 ms identified a successful RF catheter ablation from the LSV with a sensitivity of 88 %, specificity of 100%, positive predictive value of 100 % and negative predictive value of 75% [24].

### Significance of Potentials recorded at the Ablation Site within the Left Sinus of Valsalva

**Figure 4:**
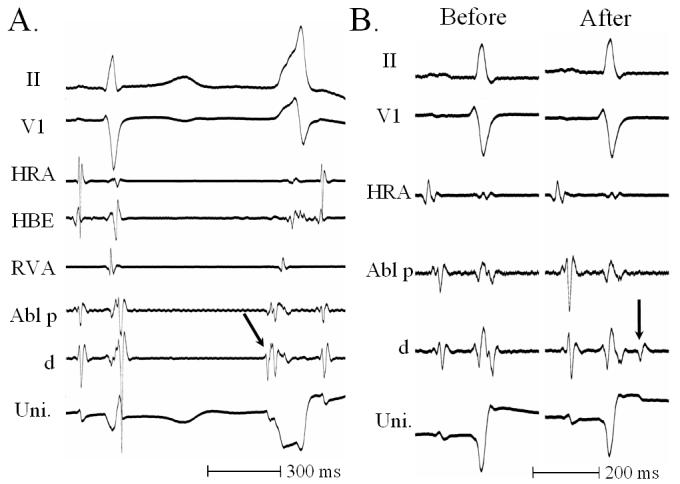
Intracardiac recordings from the successful ablation site within the left sinus of Valsalva. A. A sharp potential preceding the QRS complex (arrow) was recorded during a premature ventricular contraction (PVC). B. Intracardiac recordings during sinus rhythm. Before ablation, no potentials following the QRS complex were recorded. After the application of radiofrequency energy at this site, a potential following the QRS complex after the successful ablation (arrows) appeared during sinus rhythm. Abl= ablation catheter; Bi.= bipolar recording; d= distal; HRA= high right atrium; HBE= His bundle electrogram; p= proximal; Uni.= unipolar recording.

It is well known that a potential preceding the QRS complex is often recorded at the ablation site within the LSV (Figure 4A) [8,12,13,;25]. However, it is unclear if complete elimination of Epi-VT/PVCs can always be achieved with an application of RF energy at the site where this potential is recorded. In a recent study in which a quantitative analysis of the electrograms was performed in 23 patients with symptomatic Epi-VT/PVCs that underwent RF applications from the LSV, this potential was found at 19 (90%) of 21 successful ablation sites [26]. However, it was also recorded at 19 (79%) of 24 unsuccessful ablation sites, and there was no significant difference in the incidence of the potentials between the successful and unsuccessful ablation sites (p=0.5). In addition, there were no significant differences in the amplitude (p=0.12) or interval from the potential to the QRS complex (p=0.10) between the successful and unsuccessful ablation sites. Therefore, the presence of this potential may not help in identifying successful ablation sites within the LSV.

Another potential recorded in the late phase of the QRS complex during sinus rhythm in patients with Epi-VT/PVCs has been reported (Figure 4B)[8]. A recent study demonstrated that this potential was more often observed at the successful ablation sites than at the unsuccessful ablation sites before the ablation (p<0.05), and the difference between the two groups became greater after the ablation (p<0.001) [26]: In the unsuccessful ablation sites, this potential was absent (Type 1) or its location did not change before and after the ablation (Type 4). However, this potential appeared at 38% of the successful ablation sites (Type 2), and pre-existing potentials were more delayed after the ablation at 53% of the successful ablation sites (Type 3), demonstrating significant differences in the pattern of this potential between the successful and unsuccessful ablation sites (p<0.001). As a result, type 2 or type 3 patterns for this potential identified a successful ablation with a sensitivity of 100%, specificity of 92%, positive predictive value of 90% and negative predictive value of 100% [26]. Therefore, this potential is also characteristic of Epi-VT/PVCs, and may be more useful in identifying successful ablation sites than the former potential recorded during the Epi-VT/PVCs. However, the precise mechanism responsible for these two potentials recorded during the Epi-VT/PVCs (the former) or during sinus rhythm (the latter) is still unclear. There is a possibility that the analysis of the electrograms recorded at the successful and unsuccessful ablation sites in the same patient might be affected by the effects of the RF energy applications. Therefore, the significance and utility of these 2 potentials recorded within the LSV have not been sufficiently clarified.

## Conclusions

With an RF energy application from the LSV, some Epi-VT/PVCs can be abolished. However, RF catheter ablation within the LSV runs the risk of potential complications. Thus, attempting to diagnose these types of VT/PVCs non-invasively before the ablation procedure, performing detailed mapping and assessment of the electrograms obtained during the procedure, and obtaining a precise assessment of the anatomic relationships between the arrhythmia origin and coronary arteries and aortic valve, are crucial for avoiding futile RF applications from the LSV and the ensuing complications, and for successfully eliminating this type of Epi-VT/PVC.
